# Health system for maternal health – a case study from Papua, Indonesia

**DOI:** 10.1186/1471-2458-12-S2-A24

**Published:** 2012-11-27

**Authors:** Tiara Marthias, Laksono Trisnantoro

**Affiliations:** 1 Faculty of Medicine, Gadjah Mada University, Jogjakarta, Indonesia

## Background

In relation to health status, Papua Province is one of the most alarming parts of Indonesia, especially in maternal health. Papua Province maternal mortality ratio is disconcertingly higher than the national average. This within country disparity may be caused by the inequality in socioeconomic status, but perhaps more in the health system. This study aims to explore the factors within the health system associated with poor maternal outcome in Jayawijaya district, Papua, Indonesia.

## Materials and methods

A case study was conducted for Jayawijaya district, which is one the districts in Papua with high maternal deaths. Bottleneck analyses were used, using the WHO’s Health System and the Tanahashi’s health system Framework. Secondary data from available national surveys and government reports were analyzed, combined with direct observations on Jayawijaya’s health system to identify bottlenecks that may impede the success in improving maternal health.

## Results

Prominent causes for limited success in maternal health care were both in the demand and supply sides of the health system. The poor distribution of health workforce and the low utilization rate for essential obstetric intervention lowered the overall quality of service delivery. Information on health care coverage and basic epidemiology were limited and largely undocumented, leading to poor health financing allocation and health planning in general. Strong leadership, managerial skills, and political commitment are still lacking in Jayawijaya district.

## Conclusion

Jayawijaya district, and potentially Papua Province at large, still needs to improve its health system’s commodities. Lack of skilled staff, inadequate infrastructure, and poor monitoring as well as limited information system have led to the slow progress health. Strong political commitment and local leadership should be encouraged in order to improve the overall health system.

